# Visceral fat adipocytes from obese and colorectal cancer subjects exhibit distinct secretory and ω6 polyunsaturated fatty acid profiles and deliver immunosuppressive signals to innate immunity cells

**DOI:** 10.18632/oncotarget.10998

**Published:** 2016-08-01

**Authors:** Manuela Del Cornò, Massimo D'Archivio, Lucia Conti, Beatrice Scazzocchio, Rosaria Varì, Gloria Donninelli, Barbara Varano, Stefania Giammarioli, Simone De Meo, Gianfranco Silecchia, Francesco Pennestrì, Roberto Persiani, Roberta Masella, Sandra Gessani

**Affiliations:** ^1^ Departments of Hematology, Oncology and Molecular Medicine, Istituto Superiore di Sanità, Rome, Italy; ^2^ Departments of Veterinary Public Health and Food Safety, Istituto Superiore di Sanità, Rome, Italy; ^3^ Department of Medical-Surgical Sciences and Biotecnologies, Sapienza University of Rome, Rome, Italy; ^4^ General Surgery Unit, Catholic University, Rome, Italy

**Keywords:** obesity, adipose tissue, fatty acids, immune cells, colorectal cancer

## Abstract

Obesity is a low-grade chronic inflammatory state representing an important risk factor for colorectal cancer (CRC). Adipocytes strongly contribute to inflammation by producing inflammatory mediators. In this study we investigated the role of human visceral fat adipocytes in regulating the functions of innate immunity cells. Adipocyte-conditioned media (ACM) from obese (*n* = 14) and CRC (lean, *n* = 14; obese, *n* = 13) subjects released higher levels of pro-inflammatory/immunoregulatory factors as compared to ACM from healthy lean subjects (*n* = 13). Dendritic cells (DC), differentiated in the presence of ACM from obese and CRC subjects, expressed elevated levels of the inhibitory molecules PD-L1 and PD-L2, and showed a reduced IL-12/IL-10 ratio in response to both TLR ligand- and γδ T lymphocyte-induced maturation. Furthermore, CRC patient-derived ACM inhibited DC-mediated γδ T cell activation. The immunosuppressive signals delivered by ACM from obese and CRC individuals were associated with a pro-inflammatory secretory and ω6 polyunsaturated fatty acid profile of adipocytes. Interestingly, STAT3 activation in adipocytes correlated with dihomo-γlinolenic acid content and was further induced by arachidonic acid, which conversely down-modulated PPARγ. These results provide novel evidence for a cross-talk between human adipocytes and innate immunity cells whose alteration in obesity and CRC may lead to immune dysfunctions, thus setting the basis for cancer development.

## INTRODUCTION

White adipose tissue (AT) is increasingly recognized as a complex immunocompetent organ that integrates endocrine, metabolic and inflammatory signals. This tissue produces many bioactive molecules which not only serve as regulators of systemic metabolism, but also possess immune-regulatory properties [1]. Visceral AT is immunologically dynamic and contains, besides adipocytes, a unique immune cell repertoire [2].

Obesity disrupts the dynamic role of adipocytes in maintaining energy homeostasis by altering the metabolic and endocrine functions of these cells, thus leading to metabolic disease [3]. Increased release of fatty acids (FA) and hormones, reduction in lipid turnover, and unbalanced secretion of pro-versus anti-inflammatory mediators in AT take place in obesity [4]. Likewise, the relative proportions of AT resident immune cells is markedly altered in obesity, changing the tissue milieu, and contributing to the complex inflammatory network [5]. While in lean subjects resident immune cells have housekeeping functions contributing to apoptotic cell clearance, extracellular matrix remodeling and angiogenesis, obesity provides metabolic danger signals and drives a shift toward an inflammatory response by affecting immune cell number and phenotype [2]. Among immune cells, dendritic cells (DC) are critical sentinels of the immune system and have the unique ability to integrate a wide array of incoming signals and convey them to lymphocytes. These cells are endowed with a remarkably high functional plasticity allowing the critical decision between immune activation or tolerance [6].

Disruption of the cross-talk between adipocytes and resident leukocytes contributes to obesity-associated low-grade chronic inflammation, a primary cause of obesity-induced pathologies [7]. Enhanced adiposity is associated with increased cancer incidence, representing an important indicator of survival, prognosis and recurrence rates in several tumors including colorectal cancer (CRC) [8]. Obese subjects have a 1.5–3.5-fold increased risk of developing CRC as compared with lean individuals. Abdominal rather than total adiposity is a strong risk factor for CRC [9]. The low-grade chronic inflammation characterizing obesity, and the anti-inflammatory drug benefits in lowering CRC risk and retarding intestinal tumors in ulcerative colitis patients, provide compelling evidence for a link among inflammation, obesity and cancer [10]. Chronic inflammation contributes to cancer development via multiple mechanisms. One potential mechanism is that pro-inflammatory mediators can generate an immunosuppressive microenvironment (e.g. infiltration of immune suppressor cells, activation of immune checkpoint pathways in effector T cells) more suitable for tumor establishment and progression.

Notably, dietary components influence inflammation, and dietary habits have been suggested to play a major role in CRC risk [11]. It has only recently gained acceptance that FA are major determinants in inflammation [12] and may influence the risk of related pathologies [13, 14]. FA are key components of AT and their profile in the tissue closely reflects the dietary intake and/or innate metabolic differences [15]. The type of response they induce strongly depends on their biochemical properties, mainly number and position of double bonds [16]. Owing to the proposed competitive role of ω3 and ω6 polyunsaturated FA (PUFA), exerting anti- or pro-inflammatory activity, respectively, their dietary composition has been suggested to be a biologically plausible target for CRC prevention [17]. Remarkably, the pro-inflammatory microenvironment in AT may depend not only on positive energy balance but also on the quality of FA composition [18].

In this study we investigated the role of adipocyte microenvironment in the modulation of innate immunity cell functions. We report that, in obesity and CRC conditions, adipocyte microenvironment delivers immunosuppressive signals to differentiating DC, as assessed by their enhanced expression of inhibitory molecules and the reduced IL-12/IL-10 ratio. Likewise, DC generated in the presence of adipocyte-conditioned medium (ACM) from obese and CRC subjects exhibit an impaired capacity to functionally interact with γδ T lymphocytes. Of note, adipocytes from obese and CRC subjects were found to release higher amounts of pro-inflammatory and immunoregulatory cytokines/chemokines (IL-6, CXCL8, CCL2, IL-10) with respect to lean healthy subjects. Furthermore, distinct profiles of individual ω6 PUFA were found in adipocytes from obese and CRC subjects, independently of body mass index (BMI), in comparison to healthy lean subjects. Interestingly, adipocyte treatment with ω6 arachidonic acid (AA) affected the balance between pro- and anti-inflammatory factors promoting STAT3 activation and inhibiting PPARγ expression.

All together these results provide evidence for a cross-talk between human adipocytes and innate immunity cells which is dysregulated in obese and CRC subjects. Obesity and CRC induced alterations of adipocyte microenvironment may set the basis for cancer development and progression by altering DC function and subverting immune surveillance.

## RESULTS

### Obesity and CRC affect adipocyte immune mediator profile

**Figure 1:**
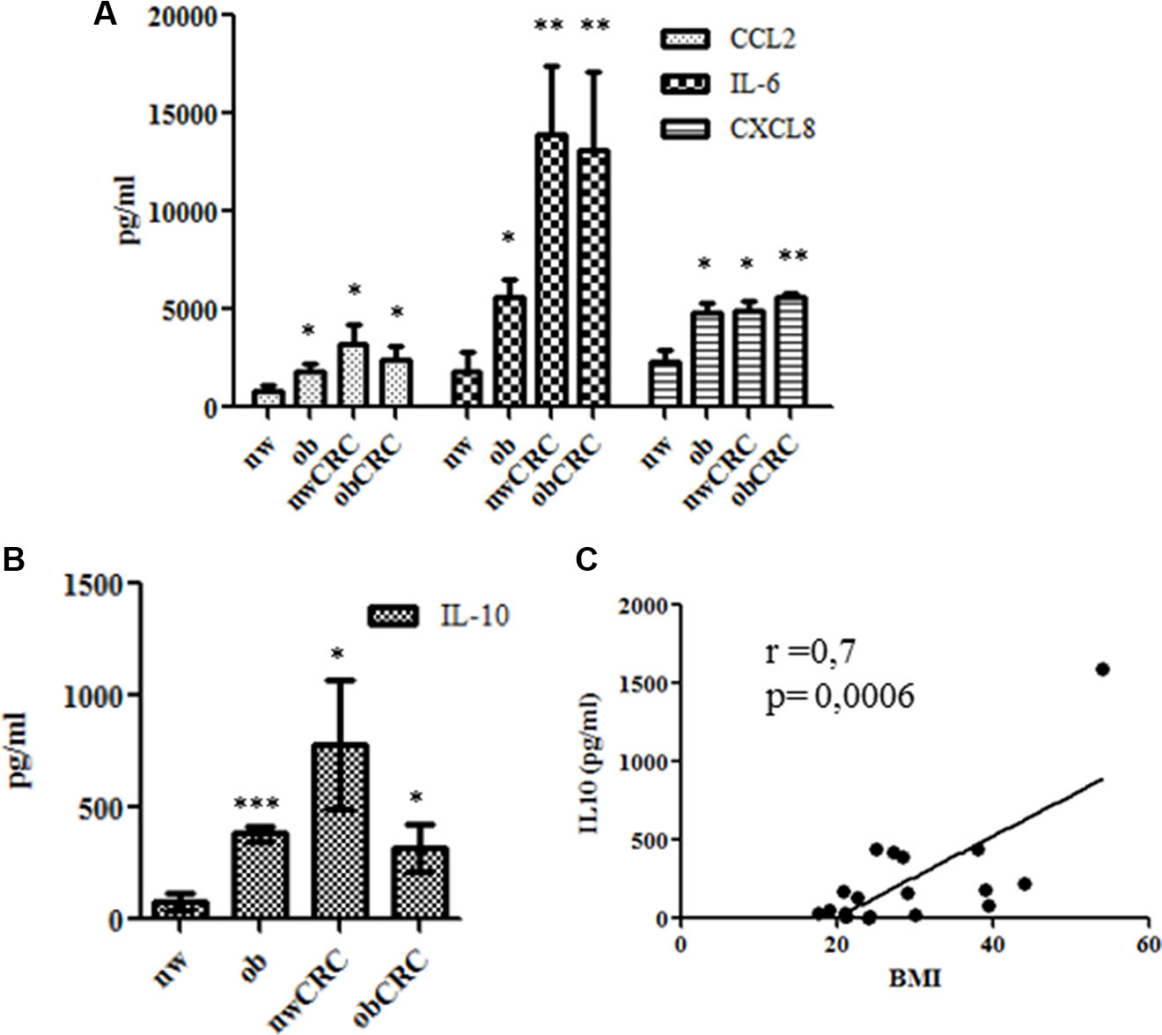
Profile of immune mediators in adipocyte microenvironment ACM were collected from lean and obese subjects, affected or not by CRC after 24 hours of culture. The content of IL-6, CXCL8, CCL2 (**A**) and IL-10 (**B** and **C**) was assessed by ELISA. Data are expressed as means ± SEM. nw (*n* = 11) vs ob (*n* = 10), nwCRC (*n* = 8) and obCRC (*n*= 8). (C) Correlation of BMI to IL-10 levels. *p values* were calculated by two-tailed unpaired Student's *t* test or Pearson's test and statistical significance is indicated. *p < 0.05; **p < 0.01; ***p < 0.001.

Several studies have reported alterations in the secretory activity of AT in obese subjects suggesting that different profiles of immune mediators might exist under obesity conditions. To investigate whether altered adipocyte soluble mediator profiles could be associated with obesity and CRC, a preliminary screening of factors (cytokines, chemokines and growth factors) released in ACM was performed by multiplex immunoassay. Among the several cytokines (IL-6, IL-10, TNF-α, G-CSF, GM-CSF, IL-1RA) and chemokines (CCL2, CCL4, CCL5, CXCL8, CXCL10) found to be secreted (data not shown), we selected a panel of immunoregulatory factors including IL-6, CXCL8, CCL2 and IL-10 for further analysis. The content of these immune mediators was thus evaluated by ELISA in supernatants of adipocytes isolated from lean and obese subjects, affected or not by CRC. As shown in Figure 1A, the expression levels of IL-6, CXCL8 and CCL2 were significantly higher in ACM from obese subjects as compared to normal weight. Interestingly, a significant increase of all these factors was also observed in ACM from CRC patients, independently of the obesity condition (Figure 1A). Specifically, IL-6 levels showed a trend toward a further increase under CRC conditions. Concomitantly to the enhanced secretion of these pro-inflammatory mediators, the release of IL-10 was also significantly increased in obese and CRC subjects (Figure 1B). Interestingly, IL-10 expression positively correlated with BMI when subjects not affected by CRC were considered (Figure 1C).

### Adipocyte microenvironment from obese and CRC subjects drives the differentiation of immunosuppressive DC

**Figure 2:**
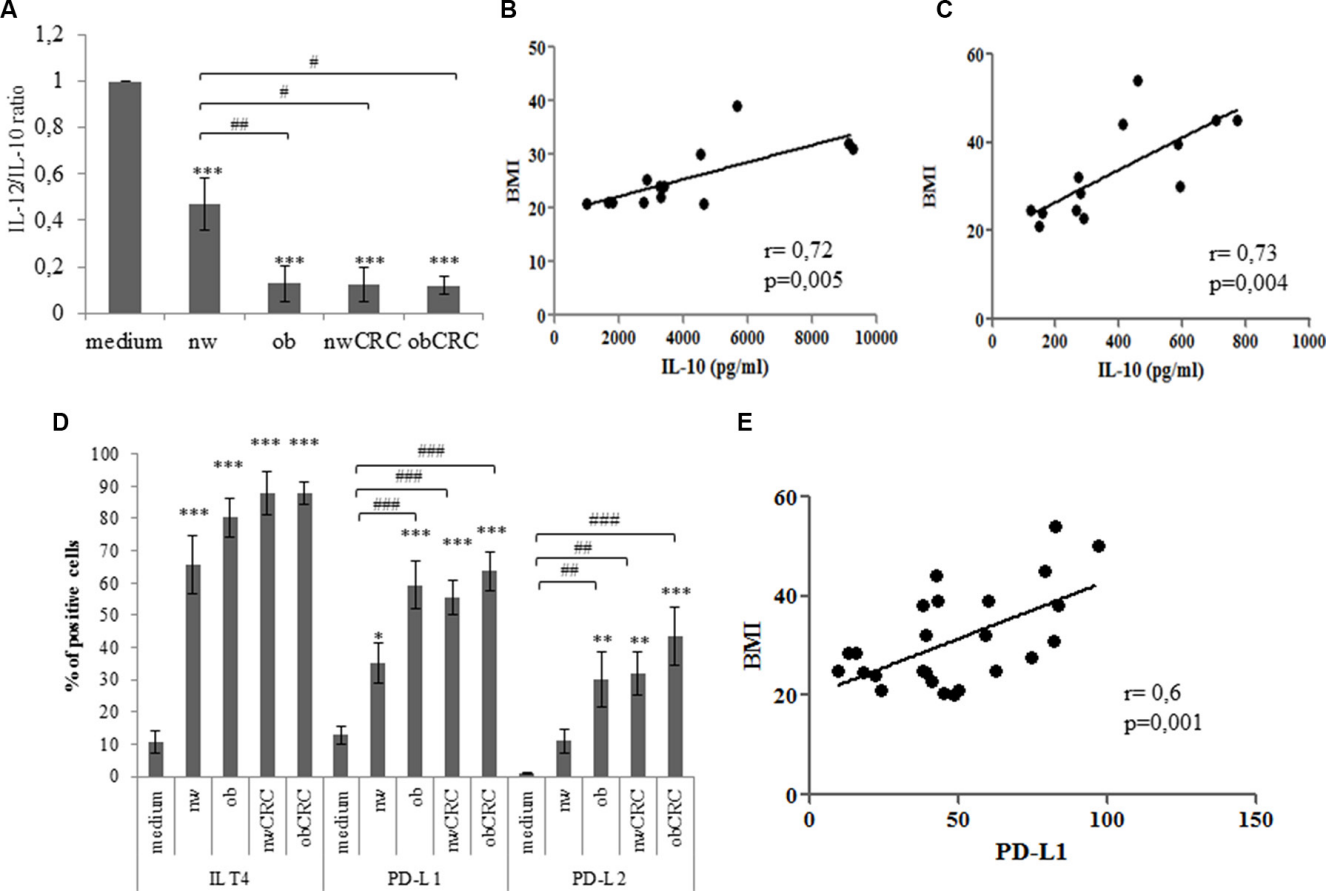
Adipocyte microenvironment from obese and CRC subjects exerts immunosuppressive effects on DC (**A**) 5-day-cultured control DC (medium), nw (*n* = 12), ob (*n* = 14), nwCRC (*n* = 8) and obCRC (*n* = 8) -ACM DC were treated with LPS (10 ng/ml). Twentyfour hours later supernatants were collected and analyzed by ELISA for IL-12 and IL-10 content. Data represent the variations in the IL-12/IL-10 ratio with respect to control DC cultures and are expressed as mean ± SEM. (**B** and **C**) Positive correlation of BMI to IL-10 levels in high (*n* = 13) and low (*n* = 13) producing DC, respectively. (**D**) 5-day-cultured control DC (medium), nw (*n* = 12), ob (*n* = 14), nwCRC (*n* = 14) and obCRC (*n* = 13) -ACM DC were analyzed by flow cytometry for the expression of the indicated surface markers. Data are shown as means of the percentage of positive cells ± SEM. (**E**) Positive correlation of BMI to PD-L1 levels (*n* = 26). *p* values were calculated by ANOVA or Pearson's test and statistical significance is indicated. (ACM DC vs medium: *p < 0.05; **p < 0.01; ***p < 0.001. nw-ACM DC vs ob-, nwCRC- and ObCRC- ACM DC: ^#^*p* < 0.05; ^##^*p* < 0.01; ^###^*p* < 0.001).

The impact of obesity and cancer on myeloid DC number and function has been described in several reports to date [19–21]. While many studies highlighted the importance of DC dysfunctions in several tumor models, relatively little is known on the influence of adipocyte milieu in determining the immunologic behavior of these cells in obesity and cancer. To evaluate whether adipocyte microenvironment could influence DC differentiation, monocytes were stimulated to differentiate towards DC with GM-CSF and IL-4 in the presence or in the absence of ACM from lean and obese subjects, affected or not by CRC. As shown in Table 1, ACM-generated DC (ACM-DC) exhibited a different phenotype with respect to DC generated under standard conditions. These cells, independently of the category of ACM, expressed CD1a, but retained high levels of CD14. Furthermore, ACM-DC expressed DC-SIGN and mannose receptor (MR), two important markers associated with DC functional activity. However, within the normal weight category of subjects, regardless of CRC presence, a lower expression of CD1a was observed with respect to the obese category affected or not by CRC. We then investigated whether ACM-DC exhibited different functional activities by assessing their phenotype and secretory profile upon maturation induction. Although these cells acquired a mature phenotype upon LPS-induced activation, as assessed by upregulation of HLA-DR and co-stimulatory molecules (Supplementary Figure S1), a significant reduction of the IL-12/IL10 ratio was found in obese with respect to lean ACM-DC (Figure 2A). Likewise, a reduced IL12/IL-10 ratio was also detected in supernatants of ACM-DC from CRC patients, independently of BMI (Figure 2A). Due to the marked variability among donors in the IL-10 levels produced by DC, data were normalized to control DC cultures and expressed as variations with respect to control. Specifically, we distinguished between low (< 1000 pg/ml, 50% of donors) and high (> 1000 pg/ml, 50% of donors) IL-10 producing DC. Despite this variability, a positive correlation was found between IL-10 levels and BMI in both groups of donors when subjects not affected by CRC were considered (Figure 2B and 2C). These results suggested that both obesity and CRC can shape adipocyte microenvironment toward immunosuppressive functions. Thus, to further characterize ACM-DC, we focused on the analysis of tolerogenic/inhibitory molecules. As shown in Figure 2D, a significant induction of PD-L1 and PD-L2 distinguished cells generated in the presence of ACM from obese and CRC patients from those obtained with ACM from lean individuals. Conversely, ILT4 was found to be equally induced in all ACM-DC types as compared to control DC (Figure 2D). Of note, a significant correlation was found between PD-L1 expression and BMI in healthy obese and lean subjects (Figure 2E).

**Table 1:** Adipocyte microenvironment modulates DC phenotypic profile

Markers	% of positive cells				
	medium	nw	ob	nwCRC	obCRC
CD14	3.8 ± 2.6	59.1 ± 10***	56.5 ± 7.5***	49.3 ± 10**	50.4 ± 16.5**
CD1a	66.7 ± 4.2	38.2 ± 6.5**^#^^#^	63.5 ± 5,3	37.6 ± 7.4**^#^^°^	61.5 ± 6.1
MR	68.2 ± 8.7	83.4 ± 14.9	67.1 ± 7.9	79.6 ± 3.2	78.3 ± 4.9
DC-SIGN	84.4 ± 4.1	82.7 ± 6.5	75.4 ± 9	82.3 ± 2.3	88.4 ± 5

5-day-cultured control DC (medium), nw (*n* = 8), ob (*n* = 13), nwCRC (*n* = 8) and obCRC (*n* = 7) -ACM DC were collected, stained with FITC-conjugated Ab to CD14 or PE-conjugated Ab to CD1a, DC-SIGN and MR and analyzed by flow cytometry. Data are shown as percentage of positive cells and represent means ± SEM. *p* values were calculated by ANOVA and statistical significance is indicated. (ACM DC vs medium:

** *p* < 0.01;

*** *p* < 0.001 or nw-ACM DC vs ob-, nwCRC- and obCRC-ACM DC:

# *p* < 0.05; nwCRC-ACM DC vs obCRC-ACM DC:

° *p* < 0.05).

### Adipocyte microenvironment from obese and CRC subjects alters the reciprocal activating interaction between DC and γδ T lymphocytes

**Figure 3:**
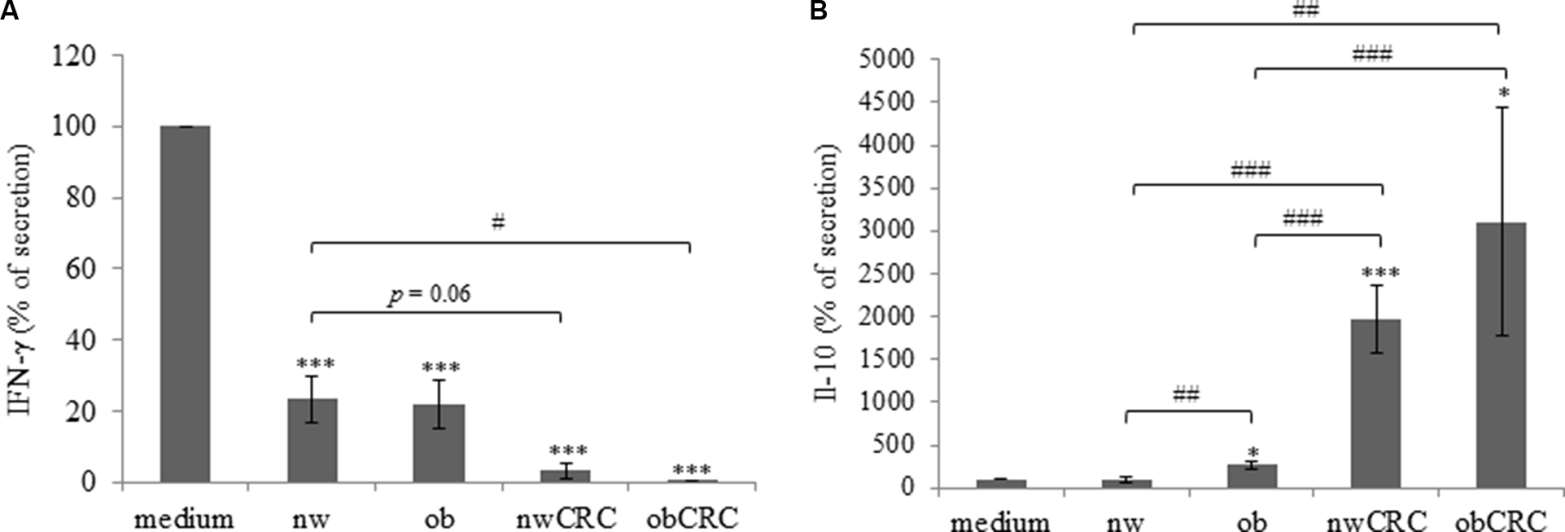
Adipocyte microenvironment from obese and CRC subjects impairs the DC/γδ T cell cross-talk 5-day-cultured control DC (medium) or nw (*n* = 11), ob (*n* = 14), nwCRC (*n* = 7) and obCRC (*n* = 7) -ACM DC were co-cultured with autologous γδ T lymphocytes (1:1 ratio) in the presence of zoledronate (10 μg/ml) for 48 hours. Supernatants were then collected and analyzed by ELISA for IFN-γ (**A**) or IL-10 (**B**) determination. Data are expressed as percentage of secretion relative to control DC co-cultures (medium). Mean ± SEM is shown for each subject group. *p* values were calculated by two-tailed unpaired Student's *t* test and statistical significance is indicated (ACM-DC vs medium: *p < 0.05; ***p <0.001. nw-ACMDC vs Ob-, nwCRC- and ObCRC- ACM DC: ^#^*p* < 0.05; ^##^*p* < 0.01; ^###^*p* < 0.001).

We have previously described that DC induce the activation of phosphoantigen-stimulated γδ T lymphocytes and these cells reciprocally promote the maturation of DC, thus enhancing their capacity to stimulate adaptive αβ T cell mediated responses [22, 23]. To investigate whether adipocyte microenvironment could affect the DC-γδ Τ cell cross-talk, control and ACM-DC were co-cultured with autologous antigen-stimulated γδ T lymphocytes and the activation of both cell populations was measured 48 hours later by assessing cytokine secretion in the co-cultures. As compared to control DC, ACM-generated cells exhibited a reduced capacity to induce γδ T cell activation, as assessed by IFN-γ production, independently of the subject category (Figure 3A), suggesting that, in homeostatic condition, adipocyte microenvironment exerts a control on the activation of this lymphocyte subset. The effect of obese ACM-DC was comparable to that exerted by normal weight ACM-generated cells. Interestingly, when DC were generated with ACM from CRC subjects, γδ T cell activation was further reduced (Figure 3A). To test the functional response of ACM-DC following their interaction with activated γδ T lymphocytes, the production of IL-12 and IL-10 in co-culture supernatants was analyzed. We observed that IL-12 release by ACM-DC was significantly reduced with respect to control cells (fold change 10), and no difference was found among donor categories (data not shown). In contrast, the production of IL-10 in the co-cultures was strongly affected when DC were generated with ACM from obese and CRC patients, whereas no effect was exerted by ACM from normal weight subjects (Figure 3B). In keeping with the reduced lymphocyte activation promoted by ACM-DC from CRC patients, higher levels of IL-10 were released in these cultures with respect to those from healthy lean and obese subjects.

### Distinct profiles of individual ω6 PUFA characterize obese and CRC individuals

We have previously demonstrated the existence of a pro-inflammatory microenvironment in adipocytes of CRC patients, as assessed by up-regulation of STAT3 activity and down-regulation of PPARγ content. Adipocyte inflammatory status was independent of obesity degree but was associated with a decreased ω3/ω6 PUFA ratio. Furthermore, an increase in ω6 PUFA in adipocytes from obese subjects with or without CRC, with respect to lean individuals has been found [18]. We thus evaluated whether the pro-inflammatory status of adipocytes found in obesity and CRC was associated with distinct signatures of individual PUFA, focusing on the ω6 family. As shown in Table 2, gas-chromatography analysis of adipocyte FA content showed no significant variations in linoleic acid (LA) level among the different categories of subjects, despite a trend toward increase was observed in obesity, independently of CRC. However, a clear-cut decrease in γlinolenic acid (GLA), exhibiting anti-inflammatory activity, was observed in adipocytes from obese and CRC subjects, paralleling a considerable increase of the pro-inflammatory AA. Likewise, AA precursor dihomo-γlinolenic acid (DGLA) was increased in CRC affected patients. In addition, a marked increase of adrenic acid (AdA), the downstream product of AA, was observed in obese and CRC subjects with respect to lean individuals, statistically significant in CRC subjects. These results indicate that in adipocytes of obese and CRC subjects, a dysregulation of the ω6 PUFA metabolic pathway leading to AA and AdA from LA occurs, suggesting its contribution to the generation of the inflammatory milieu. A simplified schematic representation of this ω6 PUFA pathway is shown in Supplementary Figure S2.

**Table 2:** Individual ω6 PUFA content of adipocytes from normal weight and obese subjects affected or not by CRC

ω6 PUFA	nw (*n* = 13)	ob (*n* = 14)	nwCRC (*n* = 13)	obCRC (*n* = 14)	*p* ANOVA
**Linoleic**	11.395 + 2.339	12.265 + 2.723	10.667 + 1.961	12.799 + 2.681	0.127
**γlinolenic**	0.068 + 0.015	0.051 + 0.016*	0.053 + 0.018*	0.052 + 0.017*	0.034
**Eicosadienoic**	0.201 + 0.040	0.191 + 0.056	0.210 + 0.038	0.238 + 0.054	0.072
**Dihomo-γlinolenic**	0.146 + 0.026	0.173 + 0.050	0.196 + 0.047*	0.203 + 0.063*	0.017
**Arachidonic**	0.245 + 0.032	0.298 + 0.056*	0.302 + 0.089	0.309 + 0.064*	0.038
**Adrenic**	0.122 + 0.030	0.179 + 0.092	0.200 + 0.086*	0.221 + 0.077**	0.010

The content of individual ω6 PUFA was evaluated in visceral adipocytes isolated from nw, ob, nwCRC, and obCRC subjects. Data are shown as percentage of total fatty acid content and represent means ± SEM. Differences among the four groups were evaluated by ANOVA and *p* value is indicated.

* *p* < 0.05 compared with nw;

** *p* < 0.01 compared with nw.

### Adipocyte inflammatory status is modulated by arachidonic acid

**Figure 4:**
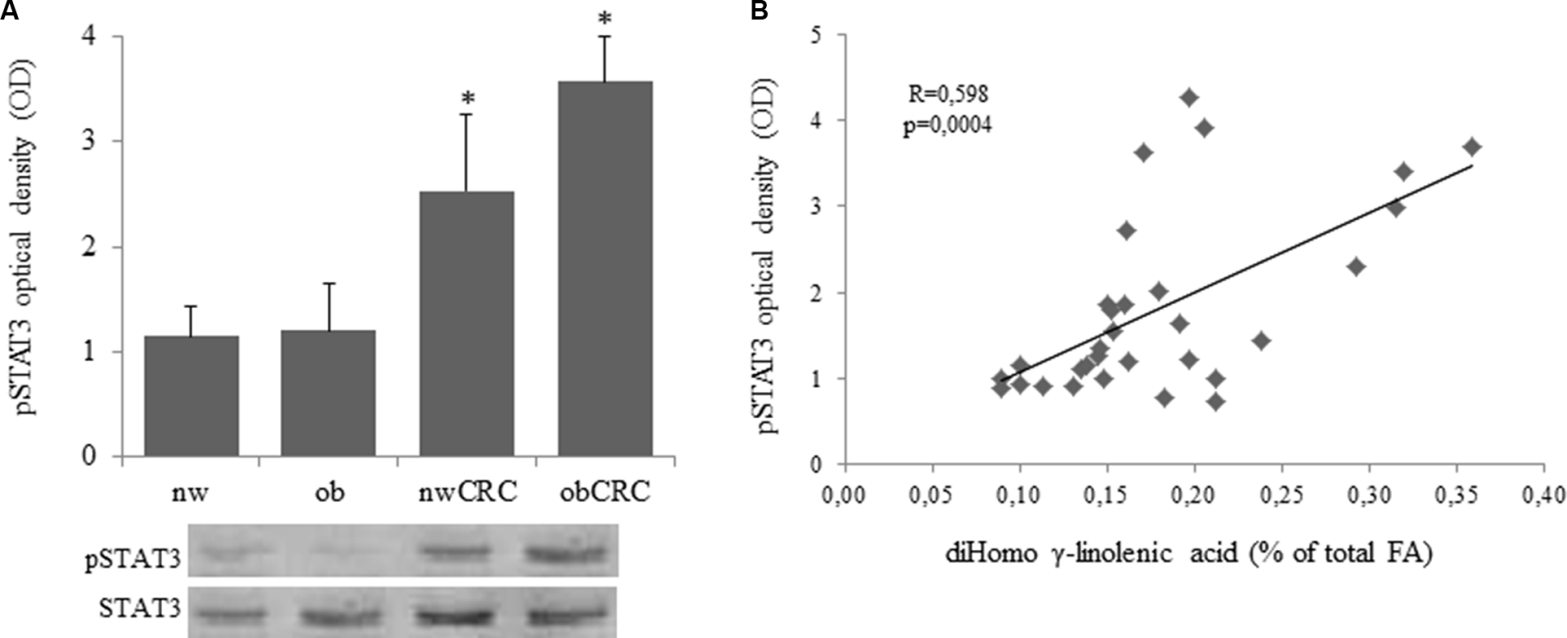
Over-expression of phospho-STAT3 in adipocytes from CRC patients and correlation with DGLA content (**A** and **B**): Human visceral adipocytes, collected from the four groups of subjects, were serum-starved for 18 hours. Whole cell extracts were separated by SDS-PAGE and analyzed using anti-phospho-STAT3 (pSTAT3) antibody. Results were normalized to STAT3 protein content and expressed as optical density (OD), and representative blots are shown. Mean ± SEM is shown for each subject group. Differences among the four groups were evaluated by ANOVA. *p < 0.01, nwCRC and ObCRC vs nw or ob.

**Figure 5:**
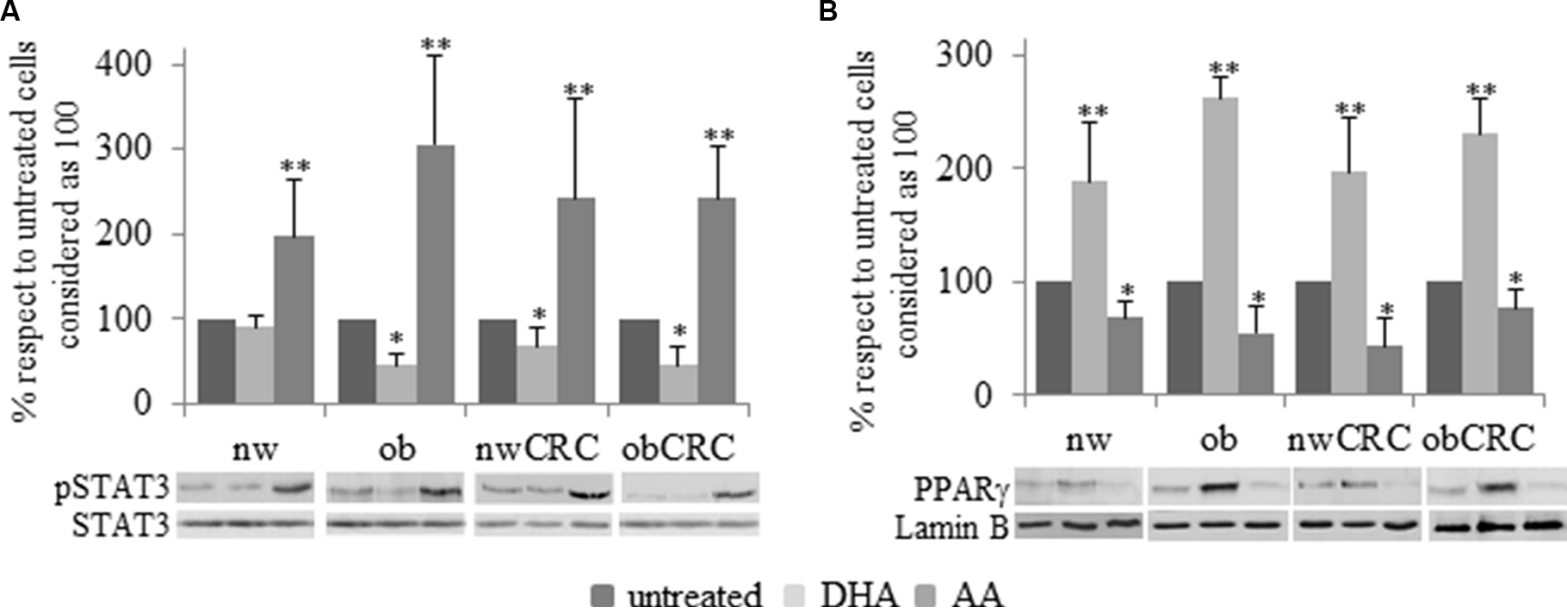
Immunoblotting analysis of pSTAT3 and PPARγ after PUFA treatment Human visceral adipocytes, collected from the four groups of subjects, were serum-starved for 18 hours and incubated with DHA or AA. Western blot analysis of pSTAT3 (**A**) and PPARγ (**B**) were carried out, and results were normalized to STAT3 and Lamin B protein content, respectively. Representative blots are shown. Mean ± SEM is shown for each subject group. Differences among each group were evaluated by ANOVA. *p < 0.05 and **p < 0.01 compared with untreated cells.

We have previously demonstrated that exogenous addition of the anti-inflammatory ω3 PUFA docosahexaenoic acid (DHA) to adipocytes of obese and CRC subjects attenuates their inflammatory status by upregulating nuclear PPARγ, the master regulator of mature adipocyte genes, and downregulating STAT3 activation [18]. In this study we found that a constitutively activated STAT3 is detected in adipocytes from CRC patients, independently of BMI, with respect to normal weight and obese groups. (Figure 4A). Moreover, correlation analysis showed that the activation status of this transcription factor positively correlates with the content of DGLA (Figure 4B). On the basis of the enhanced AA content found in obesity and CRC, we therefore investigated whether this pro-inflammatory PUFA might affect the activation of STAT3 and PPARγ. As shown in Figure 5, exposure of adipocytes to AA resulted in a significant up-regulation of phospho-STAT3 (Figure 5A) and a concomitant down-regulation of PPARγ expression (Figure 5B) in each category of subjects as compared to the untreated paired individuals. As expected, DHA exerted the opposite effect (Figure 5A and 5B).

## DISCUSSION

Excessive adiposity is associated with both increased risk of multiple malignancies and worse outcomes after diagnosis. The increasing rate of obesity worldwide is thus predicted to be associated with a surge in diseases [24]. Multiple molecular changes arising as a consequence of increased body mass are likely to contribute to the increased incidence of neoplasia and worse outcomes in obesity. Noteworthy, not only increased adipose burden but also AT inflammation has been reported in obese individuals and mouse models of obesity [25]. Moreover, increased levels of circulating pro-inflammatory mediators characterize the obese condition as a consequence of local tissue inflammation.

Several studies described alterations of AT secretome in human obesity highlighting the importance of some cytokines/chemokines not only in the establishment/perpetuation of inflammation but also in tumor initiation/progression [26–28] and metastasis promotion [29, 30]. However, little is known on the type of immune mediators released by purified adipocytes from human visceral AT and on their role in shaping immune cell profiles. We report herein that adipocytes from both obese and CRC subjects release enhanced amount of pro-inflammatory cytokines/chemokines such as IL-6, CXCL8 and CCL2. In particular, in CRC patients, independently of BMI, a further increased secretion of IL-6 was noted as compared to obese subjects. In keeping with this finding, we also demonstrated that the IL-6 inducer STAT3 is constitutively activated in adipocytes from CRC individuals. Interestingly, IL-6 levels are elevated in obesity and positively correlate with BMI [31], and a role for IL-6 in tumorigenesis has been demonstrated in animal models [32]. Likewise, chemokines and cytokines are extensively produced in the tumor microenvironment regardless of the initial triggers [33], and mediators such as IL-1β, TNFα, CCL2 and IL-6 directly promote tumor progression in experimental models of CRC [26]. More recently, CXCL8, VEGF and Pentraxin3 circulating levels were associated with increased risk of disease recurrence in CRC [34].

In the present study we also report that obesity and CRC conditions are associated with enhanced secretion of the immunosuppressive cytokine IL-10 by adipocytes, and we provide the first evidence that IL-10 content positively correlates with BMI. Interestingly, both IL-6 and IL-10 ARE STAT3 inducers and might be involved in its constitutive activation in adipocytes from CRC patients.

Growing evidence highlights the importance of adipocyte “secretome” in modulating the function of several cell types including immune and cancer cells. Adipocyte-secreted factors can act both as regulators of immune cell functions [35, 36] by driving the generation of suppressor cells [37], and as promoters of epithelium disruption and tumor cell proliferation/migration [38]. Of note, current evidence suggests that DC can exist in a multitude of functional states and that the immuno-stimulatory capacity of these cells is conditioned by the microenvironment [39]. This suggests that low-grade chronic inflammation associated with obesity generates an AT microenvironment that may favor cancer cell growth either directly or by suppressing immune response and surveillance. In this regard, several pathologies that differ in their etiology and physiology (e.g. autoimmune and infectious diseases) are characterized by immunosuppression, and chronic inflammation, which is shared by these diseases, is responsible for impaired immune responses [40].

We provide novel evidence that the adipocyte microenvironment in obesity and CRC affects DC differentiation and functional activities by delivering immunosuppressive signals. ACM from obese and CRC subjects drive the generation of DC expressing high levels of the inhibitory molecules PD-L1 and PD-L2, as compared to ACM from lean individuals. Interestingly, both IL-6 and IL-10 have been reported to regulate the expression of PD-L1 in DC [41]. Notably, PD-L1 and PD-L2 are involved in tumor immunosuppression and represent key immune checkpoints in targeted cancer therapy.

We also show that ACM-DC from obese and CRC subjects exhibit an altered IL-12/IL-10 ratio when stimulated to mature by either TLR ligands (i.e. LPS) or γδ T lymphocytes. As a high IL-12/IL-10 ratio has been associated with the capacity of antigen presenting cells to stimulate T cell responses and IFN-γ production, our results suggest that obese and CRC adipocyte microenvironment might affect the immuno-stimulatory properties of DC with possible alteration in T cell mediated immunity. In keeping with this hypothesis, we report for the first time that ACM-DC from CRC patients exhibit a reduced capacity to induce IFN-γ production in γδ T cells. Owing to the role of γδ T cells and of Th1 protective responses in tumor immune surveillance, our findings suggest that the adipocyte-induced alterations in innate immunity cell populations may be detrimental for the control of tumor growth. In this regard, it is interesting to note that tumor cells develop mechanisms to escape from immune responses through DC suppression, and that tumor microenvironment from both early and late-staged CRC abrogates LPS-induced IL-12 secretion in these cells while increasing IL-10 [42].

The precise immunoregulatory role of AT as well as of its influence on myeloid cell differentiation and function in obesity has not been extensively studied. However, evidence has been achieved that obesity is responsible For numerical and functional impairment of DC both in peripheral blood and locally in AT infiltrating cells [43, 44]. In keeping with these observations, we report a positive correlation between BMI and inhibitory DC regulators (i.e. IL-10 and PD-L1).

On the basis of our results and data from the literature, it is reasonable to hypothesize that factor(s) differentially released by adipocytes (e.g. cytokines/chemokines, lipid mediators, other still undefined factors) in homeostasis or in pathological conditions may differently affect DC biology. In this regard, few studies have reported the influence of ACM on the differentiation/function of other immune cells such as macrophages and T lymphocytes [35, 36, 45, 46] highlighting that, in addition to the proteic factors, the lipid fraction of ACM plays an important role.

Among the different factors potentially influencing the adipocyte microenvironment, FA composition most likely plays a pivotal role since FA can exert pro- or anti-inflammatory activities. In particular, ω3 PUFA such as DHA and eicosapentanoic acid, mainly found in seafood, exert beneficial effects including prevention of cancer and cardiovascular diseases [47, 48]. Our previous observations have demonstrated that the ω3/ω6 PUFA ratio significantly decreases in obese and CRC subjects with respect to lean individuals, and negatively correlates with BMI. In addition, adipocyte ω6 PUFA content increases in obese subjects, especially when affected by CRC [18]. In this study, we extended these findings by assessing specific PUFA belonging to the ω6 family, abundantly present in the daily diet, that can exert a variety of beneficial or detrimental effects. For instance, the downstream products of LA, GLA and its metabolite DGLA (Supplementary Figure S2), have been shown to exert anti-inflammatory and anti-tumor effects [49], while AA and AdA, also produced from DGLA, have been tightly implicated in inflammatory disorders and cancer development [50]. Our results indicate a significant decrease of the anti-inflammatory GLA, that parallels the increase of pro-inflammatory AA in adipocytes from obese subjects and of AdA and DGLA in adipocytes from CRC patients as compared to normal weight. Taken together these findings suggest that GLA is increasingly metabolized in DGLA and, eventually, in AA and AdA in obese and CRC subjects. The effective mechanisms of action of PUFA have not been fully clarified yet, however their different metabolic fate, leading to the synthesis of different classes of eicosanoids (prostaglandins, leukotrienes and isoprostanes), has been indicated as a main mechanism most likely responsible for the anti- or pro-inflammatory activities [51–53]. Furthermore, the entire picture gains further complexity since some ω6 PUFA can be the precursors of ω3 PUFA (Supplementary Figure S2). Worth of note, GLA and DGLA undergo oxidative metabolism by cyclooxygenases and lipoxygenases to produce anti-inflammatory eicosanoids (including prostaglandins of series 1 and leukotrienes of series 3) while AA and AdA give rise to the production of pro-inflammatory eicosanoids (including prostaglandins and leukotrienes of series 4) [54, 55]. Notably, prostaglandins have been demonstrated to facilitate CRC progression by stimulating cell proliferation and survival, tumor cell invasiveness and production of pro-angiogenic agents [56, 57].

In the light of our results, it is reasonable to hypothesize that a dysregulation of the metabolic pathways leading to unbalanced production of pro- and anti-inflammatory eicosanoids, occurs in obese and CRC subjects contributing to the inflamed AT microenvironment. Since AT FA profile closely reflects dietary habits, a different intake of FA among subject categories could contribute to the establishment of a pro-inflammatory milieu in which the ω6 PUFA profiles may play an important role.

The potential link between AT composition and inflammatory processes is further supported by the observed positive correlation between the level of activated STAT3 in adipocytes and their content of DGLA. Notably, treatment of adipocytes from the four groups of subjects with AA determines a significant increase in activated STAT3 levels clearly supporting a role of this ω6 PUFA in eliciting an inflammatory response. Overall, these results add further evidence for the presence of a pro-inflammatory microenvironment in AT of obese and CRC subjects sustained by distinct ω6 PUFA profiles that may reflect the inflammatory status underlying these pathological conditions. Worth of note, it has been recently published that DGLA and AA contents are higher in tumor than in normal tissues within the same CRC patient [58].

Although the factor(s) contributing to immune cell dysfunctions remain to be determined, our results provide clear-cut evidence for the existence of a cross-talk between adipocytes and innate immunity cells, as well as for its role in obesity- and CRC-associated dysfunctions. Notably, alterations of AT microenvironment associated with obesity closely resemble those found in CRC, being both pathologies characterized by inflammation. This provides further evidence that AT inflammation has a key role in carcinogenesis and that hyper-activated inflammatory pathways in adipocytes can subvert protective immune surveillance of cancers, by altering immune cell functions.

## MATERIALS AND METHODS

### Ethics statements

Investigation has been conducted in accordance with the ethical standards and with the Declaration of Helsinki, and according to national and international guidelines. It has been approved by the authors' institutional review board. All enrolled subjects were provided with complete information about the study and asked to sign an informed consent.

Healthy donor buffy coats were obtained from Centro Trasfusionale of “Sapienza” University of Rome. Buffy coats were not obtained specifically for this study. Informed consent has not been asked because data were analyzed anonymously. Data from these donors have been treated by Centro Trasfusionale according to the Italian law on personal data management “Codice in materia di protezione dei dati personali” (Testo unico D.L. June 30, 2003 n. 196).

### Patients and sample collection

Human visceral adipose tissue and blood samples were collected from age and sex matched lean and obese subject groups undergoing abdominal surgery or laparoscopy for benign (i.e. gallbladder disease without icterus, umbilical hernia, and uterine fibromatosis) or CRC conditions (histologically proved primary colon adenocarcinoma, stage Duke's A,B/stage I–II). The exclusion's criteria were: clinical evidence of active infection, recent (within 14 days) use of antibiotics/anti-inflammatory drugs, pregnancy, hormonal therapies, severe mental illness, autoimmune diseases, family history of cancer, other neoplastic diseases. Fifty-four subjects belonging to the following four groups were enrolled: normal weight (nw; *n* = 13); obese (ob; *n* = 14); normal weight with CRC (nwCRC; *n* = 14) and obese with CRC (obCRC; *n* = 13). In the normal weight groups, the BMI range was 20–25 Kg/m^2^. In the obese groups BMI was ≥ 30 Kg/m^2^, and waist circumference > 95 cm for men and > 80 cm for women. The availability of material and the use of human tissue and primary cells did not allow to perform all the analyses on the same number of subjects. For each analysis the exact number is indicated. AT sampling was performed as previously described [18]. Blood samples were drawn at the time of obtaining peripheral vein access for surgery.

### Adipocyte isolation and culture

Fifteen to forty grams of AT biopsies were microdissected, rinsed several times in 0.9% NaCl, and digested with 5 ml of Krebs-Ringer solution (0.12 M NaCl, 4.7 M KCl, 2.5 mM CaCl_2_, 1.2 mM MgSO_4_, 1.2 mM KH_2_PO_4_) containing 20 mM HEPES pH 7.4, 3.5% fatty acid-free BSA, 200 nM adenosine, 2 mM glucose and collagenase (type 1) for 1 hour (1 mg/g AT) at 37°C in shaking water bath [30]. After collagenase digestion the adipocytes were isolated and cultured in low-glucose Dulbecco's modified Eagle's medium (1,000 mg/L D-(+) -glucose) and ACM was collected after 18 hours. In some experiments, adipocytes were stimulated with DHA or AA. DHA and AA were dissolved under nitrogen condition in 100% ethanol to make 10 mM stock solutions, which were stored at −20°C. Stock solutions were diluted in culture media prior to the cell treatment. Final concentration of ethanol in treated cells was less than 0.1%. To define the lowest effective concentration of DHA (Sigma Aldrich, St. Louis, MO, USA) and AA (Cayman Chemical Company, Ann Arbor, MI USA) able to modulate adipocyte activities, we carried out preliminary experiments, incubating the isolated adipocytes with different concentration of DHA (5–50 μM) and AA (1–25 μM) for different periods of time (6–24 hours). On the basis of the data obtained (not shown), the experiments were carried out incubating the adipocytes with 10 μM DHA and 5 μM AA for 18 hours, respectively.

### Monocyte-derived dendritic cell generation and culture

Monocytes were isolated from the peripheral blood of healthy donors by Ficoll/Paque density gradient centrifugation followed by immunomagnetic selection using CD14^+^ microbeads (MACS monocyte isolation kit, Miltenyi Biotec, Auburn, CA), according to the manufacturer's instructions. To obtain control immature monocyte-derived DC, monocytes were seeded at 1 × 10^6^ cells/ml in 50% RPMI 1640 medium and 50% DMEM, containing 10% FBS, GM-CSF (50 ng/ml, kindly provided by Schering-Plough (Dardilly, France) and IL-4 (500 U/ml, Miltenyi Biotec, Auburn, CA). ACM-DC were generated in the same conditions by replacing DMEM with ACM (1:2 dilution). Fresh medium plus cytokines was added at day 3 of culture. In some experiments, on day 5, DC were stimulated with LPS (10 ng/ml) for 24 hours to obtain mature DC.

### γδ T cell isolation and co-culture with DC

γδ T lymphocytes were isolated from cryopreserved PBMC from healthy donors by positive selection with immunomagnetic beads (Miltenyi Biotec), according to the manufacturer's instructions. Briefly, PBMC were incubated with apten-conjugated anti-γδ TCR antibodies (Abs) for 15 minutes at 4°C, washed, and then incubated with FITC-conjugated anti-apten immunomagnetic beads. Positively selected population contained > 95% viable γδ T cells as assessed by flow cytometry.

After an overnight culture in complete medium (RPMI plus 10% FBS), purified γδ T cells were washed, suspended in the same medium at the density of 10^6^ cells/ml, and added to autologous control or ACM-generated DC cultures (1:1 ratio). DC/γδ T cell co-cultures were left untreated or stimulated with the nonpeptide phospho-antigen Zoledronate (ZOL, 10 μg/ml, kindly provided by Novartis Pharma, Origgio, VA) and analyzed 48 hours later.

### Fatty acids analysis

Total lipids from WAT samples were extracted with chloroform-methanol 2:1 (v/v) according to Folch et al. [59]. FA methyl esters were prepared with 2% methanolic HCl at 100°C for 2 hours, and extracted with hexane after addition of 2% sodium bicarbonate. All reagents were added with butylated hydroxy toluene (BHT; 25 mg/L) to avoid autoxidation of PUFA [60]. FA methyl esters were analyzed using a Perkin Elmer Clarus 500 gas chromatograph, as previously described [18]. Peaks were identified by comparison of their retention times with FA methyl ester standards (Supelco 37 Components FAME Mix, Sigma-Aldrich) and quantified respect to triheptadecanoin (Sigma-Aldrich) used as internal standard (IS). The individual FA detected were expressed as a percent of total FA.

### Protein determination by Western blot analysis

Whole cell extracts were prepared from adipocytes as previously described [61]. Immunoblotting analyses were carried out using antibodies specific for STAT3, the tyrosine phosphorylated form of STAT3 (pSTAT3), (Cell Signaling Technology, Danvers, MA, USA) and PPARγ, (Santa Cruz Biotechnology, Santa Cruz, CA, USA). Blots were treated with appropriate secondary antibodies conjugated with horseradish peroxidase (Santa Cruz Biotechnology) followed by ECL detection (Amersham Bio-sciences, Buckinghamshire, UK). Equal loading of proteins was verified by immunoblotting with total STAT3 or anti-Lamin B (Santa Cruz Biotechnology) antibodies. Densitometric analysis was performed using a molecular imager FX (Bio-Rad, Hercules, CA, USA).

### Evaluation of cytokine/chemokine secretion

ACM as well as supernatants from LPS-stimulated DC and DC-γδ T cell co-cultures were analyzed by ELISA for their content of IL-12, CCL2 (R&D Systems Inc, Minneapolis, MN, USA), CXCL8, IL-6, IL-10 and IFN-γ (Biolegend, San Diego, CA), according to the manufacturer's instructions. Basal levels of IL-10 in ACM kept for 5 days in the same condition of ACM-DC, were also determined, as internal control.

### Flow cytometry analysis

DC were pre-incubated for 30 minutes on ice with PBS containing 10% human AB serum to block nonspecific Ig binding and then incubated with the specific Abs or control isotypes for 30 minutes on ice, washed and analyzed. Cell staining was performed by using the following Abs: CD1a and CD14 (-PE and FITC, respectively, BD Pharmigen), ILT4 (-FITC, R&D System), PDL-1, PDL-2, MR, DC-SIGN, CD80, CD86 and HLA-DR (-PE, BD Pharmigen). Stained cells were analyzed by a FACSCalibur flow Cytometer (BD Biosciences) using the CellQuest program.

### Statistical analysis

GraphPad Prism 5 software was used for statistical analysis. Statistical comparison between groups was performed by the one-way analysis of variance (ANOVA) with Newman-Keuls post hoc test and by the two-tailed unpaired Student's *t* test for independent samples, as appropriate. Comparisons were expressed as means from several experiments ± SEM. Pearson's test was performed to determine simple correlation between two variables. Differences were considered significant when *p* values were < 0.05.

## SUPPLEMENTARY MATERIALS FIGURES



## References

[1] Cildir G, Akincilar SC, Tergaonkar V (2013). Chronic adipose tissue inflammation: all immune cells on the stage. Trends Mol Med.

[2] Schipper HS, Prakken B, Kalkhoven E, Boes M (2012). Adipose tissue-resident immune cells: key players in immunometabolism. Trends Endocrin Met.

[3] Apostolopoulos V, de Courten MP, Stojanovska L, Blatch GL, Tangalakis K, de Courten B (2016). The complex immunological and inflammatory network of adipose tissue in obesity. Mol Nut Food Res.

[4] Sam S, Mazzone T (2014). Adipose tissue changes in obesity and the impact on metabolic function. Transl Res.

[5] Grant RW, Dixit VD (2015). Adipose tissue as an immunological organ. Obesity (Silver Spring).

[6] Iwasaki A, Medzhitov R (2015). Control of adaptive immunity by the innate immune system. Nat Immunol.

[7] Exley MA, Hand L, O'shea D, Lynch L (2014). Interplay between the immune system and adipose tissue in obesity. The J Endocrinol.

[8] Khandekar MJ, Cohen P, Spiegelman BM (2011). Molecular mechanisms of cancer development in obesity. Nat Rev Cancer.

[9] Bardou M, Barkun AN, Martel M (2013). Obesity and colorectal cancer. Gut.

[10] Gregor MF, Hotamisligil GS (2011). Inflammatory mechanisms in obesity. Annu Rev Immunol.

[11] Turati F, Edefonti V, Bravi F, Ferraroni M, Talamini R, Giacosa A, Montella M, Parpinel M, La Vecchia C, Decarli A (2012). Adherence to the European food safety authority's dietary recommendations and colorectal cancer risk. Eur J Clin Nutr.

[12] Wall R, Ross RP, Fitzgerald GF, Stanton C (2010). Fatty acids from fish: the anti-inflammatory potential of long-chain omega-3 fatty acids. Nut Rev.

[13] Lindqvist HM, Sandberg AS, Fagerberg B, Hulthe J (2009). Plasma phospholipid EPA and DHA in relation to atherosclerosis in 61-year-old men. Atherosclerosis.

[14] Sun Q, Ma J, Campos H, Rexrode KM, Albert CM, Mozaffarian D, Hu FB (2008). Blood concentrations of individual long-chain n-3 fatty acids and risk of nonfatal myocardial infarction. Am J Clin nut.

[15] Pounis G, de Lorgeril M, Salen P, Laporte F, Krogh V, Siani A, Arnout J, Cappuccio FP, van Dongen M, Donati MB, de Gaetano G, Iacoviello L, European Collaborative Group of the IP (2014). Dietary patterns and fatty acids levels of three European populations. Results from the IMMIDIET study. Nut Met Cardiovas.

[16] De Caterina R, Bernini W, Carluccio MA, Liao JK, Libby P (1998). Structural requirements for inhibition of cytokine-induced endothelial activation by unsaturated fatty acids. J Lipid Res.

[17] Sasazuki S, Inoue M, Iwasaki M, Sawada N, Shimazu T, Yamaji T, Takachi R, Tsugane S (2011). Intake of n-3 and n-6 polyunsaturated fatty acids and development of colorectal cancer by subsite: Japan Public Health Center-based prospective study. Int J Cancer.

[18] D'Archivio M, Scazzocchio B, Giammarioli S, Fiani ML, Vari R, Santangelo C, Veneziani A, Iacovelli A, Giovannini C, Gessani S, Masella R (2013). omega3-PUFAs exert anti-inflammatory activity in visceral adipocytes from colorectal cancer patients. Plos One.

[19] Macia L, Delacre M, Abboud G, Ouk TS, Delanoye A, Verwaerde C, Saule P, Wolowczuk I (2006). Impairment of dendritic cell functionality and steady-state number in obese mice. J Immunol.

[20] Musilli C, Paccosi S, Pala L, Gerlini G, Ledda F, Mugelli A, Rotella CM, Parenti A (2011). Characterization of circulating and monocyte-derived dendritic cells in obese and diabetic patients. Mol Immunol.

[21] Legitimo A, Consolini R, Failli A, Orsini G, Spisni R (2014). Dendritic cell defects in the colorectal cancer. Hum Vaccin Immunother.

[22] Scotet E, Nedellec S, Devilder MC, Allain S, Bonneville M (2008). Bridging innate and adaptive immunity through gammadelta T-dendritic cell crosstalk. Front Biosci.

[23] Conti L, Casetti R, Cardone M, Varano B, Martino A, Belardelli F, Poccia F, Gessani S (2005). Reciprocal activating interaction between dendritic cells and pamidronate-stimulated gammadelta T cells: role of CD86 and inflammatory cytokines. J Immunol.

[24] Howe LR, Subbaramaiah K, Hudis CA, Dannenberg AJ (2013). Molecular pathways: adipose inflammation as a mediator of obesity-associated cancer. Clin Cancer Res.

[25] Osborn O, Olefsky JM (2012). The cellular and signaling networks linking the immune system and metabolism in disease. Nat Med.

[26] Yehuda-Shnaidman E, Schwartz B (2012). Mechanisms linking obesity, inflammation and altered metabolism to colon carcinogenesis. Obes Rev.

[27] Hursting SD, Dunlap SM (2012). Obesity, metabolic dysregulation, and cancer: a growing concern and an inflammatory (and microenvironmental) issue. Ann N Y Acad Sci.

[28] Gilbert CA, Slingerland JM (2013). Cytokines, obesity, and cancer: new insights on mechanisms linking obesity to cancer risk and progression. Annu Rev Med.

[29] Taniguchi K, Karin M (2014). IL-6 and related cytokines as the critical lynchpins between inflammation and cancer. Semin Immunol.

[30] Martinez-Useros J, Garcia-Foncillas J (2016). Obesity and colorectal cancer: molecular features of adipose tissue. J Trans Med.

[31] Kern PA, Ranganathan S, Li C, Wood L, Ranganathan G (2001). Adipose tissue tumor necrosis factor and interleukin-6 expression in human obesity and insulin resistance. American journal of physiology Endocrinol Metab.

[32] Park EJ, Lee JH, Yu GY, He G, Ali SR, Holzer RG, Osterreicher CH, Takahashi H, Karin M (2010). Dietary and genetic obesity promote liver inflammation and tumorigenesis by enhancing IL-6 and TNF expression. Cell.

[33] Mantovani A, Allavena P, Sica A, Balkwill F (2008). Cancer-related inflammation. Nature.

[34] Di Caro G, Carvello M, Pesce S, Erreni M, Marchesi F, Todoric J, Sacchi M, Montorsi M, Allavena P, Spinelli A (2016). Circulating Inflammatory Mediators as Potential Prognostic Markers of Human Colorectal Cancer. PLoS One.

[35] Ioan-Facsinay A, Kwekkeboom JC, Westhoff S, Giera M, Rombouts Y, van Harmelen V, Huizinga TW, Deelder A, Kloppenburg M, Toes RE (2013). Adipocyte-derived lipids modulate CD4 + T-cell function. Eur J Immunol.

[36] Klein-Wieringa IR, Andersen SN, Kwekkeboom JC, Giera M, de Lange-Brokaar BJ, van Osch GJ, Zuurmond AM, Stojanovic-Susulic V, Nelissen RG, Pijl H, Huizinga TW, Kloppenburg M, Toes RE (2013). Adipocytes modulate the phenotype of human macrophages through secreted lipids. J Immunol.

[37] Kennedy DE, Knight KL (2015). Inhibition of B Lymphopoiesis by Adipocytes and IL-1-Producing Myeloid-Derived Suppressor Cells. J Immunol.

[38] Coelho P, Almeida J, Prudencio C, Fernandes R, Soares R (2016). Effect of Adipocyte Secretome in Melanoma Progression and Vasculogenic Mimicry. J Cell Biochem.

[39] Chen K, Wang JM, Yuan R, Yi X, Li L, Gong W, Yang T, Su S (2016). Tissue-resident dendritic cells and diseases involving dendritic cell malfunction. Int Immunopharmacol.

[40] Baniyash M, Sade-Feldman M, Kanterman J (2014). Chronic inflammation and cancer: suppressing the suppressors. Cancer Immunol Immunother.

[41] Wolfle SJ, Strebovsky J, Bartz H, Sahr A, Arnold C, Kaiser C, Dalpke AH, Heeg K (2011). PD-L1 expression on tolerogenic APCs is controlled by STAT-3. Eur J Immunol.

[42] O'Toole A, Michielsen AJ, Nolan B, Tosetto M, Sheahan K, Mulcahy HE, Winter DC, Hyland JM, O'Connell PR, Fennelly D, O'Donoghue D, O'sullivan J, Doherty GA (2014). Tumour microenvironment of both early- and late-stage colorectal cancer is equally immunosuppressive. Brit J Cancer.

[43] Bertola A, Ciucci T, Rousseau D, Bourlier V, Duffaut C, Bonnafous S, Blin-Wakkach C, Anty R, Iannelli A, Gugenheim J, Tran A, Bouloumie A, Gual P (2012). Identification of adipose tissue dendritic cells correlated with obesity-associated insulin-resistance and inducing Th17 responses in mice and patients. Diabetes.

[44] Stefanovic-Racic M, Yang X, Turner MS, Mantell BS, Stolz DB, Sumpter TL, Sipula IJ, Dedousis N, Scott DK, Morel PA, Thomson AW, O'Doherty RM (2012). Dendritic cells promote macrophage infiltration and comprise a substantial proportion of obesity-associated increases in CD11c + cells in adipose tissue and liver. Diabetes.

[45] Guo F, He H, Fu ZC, Huang S, Chen T, Papasian CJ, Morse LR, Xu Y, Battaglino RA, Yang XF, Jiang Z, Xin HB, Fu M (2015). Adipocyte-derived PAMM suppresses macrophage inflammation by inhibiting MAPK signalling. Biochem J.

[46] Kohlstedt K, Trouvain C, Namgaladze D, Fleming I (2011). Adipocyte-derived lipids increase angiotensin-converting enzyme (ACE) expression and modulate macrophage phenotype. Basic Res Cardiol.

[47] Geelen A, Schouten JM, Kamphuis C, Stam BE, Burema J, Renkema JM, Bakker EJ, van't Veer P, Kampman E (2007). Fish consumption, n-3 fatty acids, and colorectal cancer: a meta-analysis of prospective cohort studies. Am J epidemio.

[48] Mozaffarian D, Wu JH (2011). Omega-3 fatty acids and cardiovascular disease: effects on risk factors, molecular pathways, and clinical events. J Am Coll Cardiol.

[49] Das UN (2011). Lipoxins as biomarkers of lupus and other inflammatory conditions. Lipids Health Dis.

[50] Thiebaut AC, Chajes V, Gerber M, Boutron-Ruault MC, Joulin V, Lenoir G, Berrino F, Riboli E, Benichou J, Clavel-Chapelon F (2009). Dietary intakes of omega-6 and omega-3 polyunsaturated fatty acids and the risk of breast cancer. Int J Cancer.

[51] Galano JM, Lee JC, Gladine C, Comte B, Le Guennec JY, Oger C, Durand T (2015). Non-enzymatic cyclic oxygenated metabolites of adrenic, docosahexaenoic, eicosapentaenoic and alpha-linolenic acids; bioactivities and potential use as biomarkers. Biochim Biophys Acta.

[52] Sales KJ, Katz AA, Davis M, Hinz S, Soeters RP, Hofmeyr MD, Millar RP, Jabbour HN (2001). Cyclooxygenase–2 expression and prostaglandin E synthesis are up-regulated in carcinomas of the cervix: a possible autocrine/paracrine regulation of neoplastic cell function via EP2/EP4 receptors. J Clin Endocrinol Metab.

[53] Gianetti J, De Caterina M, De Cristofaro T, Ungaro B, Guercio RD, De Caterina R (2001). Intravenous prostaglandin E1 reduces soluble vascular cell adhesion molecule-1 in peripheral arterial obstructive disease. Am Heart J.

[54] Wiktorowska-Owczarek A, Berezinska M, Nowak JZ (2015). PUFAs: Structures, Metabolism and Functions. Adv Clin Exp Med.

[55] Xu Y, Qi J, Yang X, Wu E, Qian SY (2014). Free radical derivatives formed from cyclooxygenase-catalyzed dihomo-gamma-linolenic acid peroxidation can attenuate colon cancer cell growth and enhance 5-fluorouracil's cytotoxicity. Redox bio.

[56] Stolina M, Sharma S, Lin Y, Dohadwala M, Gardner B, Luo J, Zhu L, Kronenberg M, Miller PW, Portanova J, Lee JC, Dubinett SM (2000). Specific inhibition of cyclooxygenase 2 restores antitumor reactivity by altering the balance of IL-10 and IL-12 synthesis. J Immunol.

[57] Greenhough A, Smartt HJ, Moore AE, Roberts HR, Williams AC, Paraskeva C, Kaidi A (2009). The COX-2/PGE2 pathway: key roles in the hallmarks of cancer and adaptation to the tumour microenvironment. Carcinogenesis.

[58] Yang K, Li H, Dong J, Dong Y, Wang CZ (2015). Expression profile of polyunsaturated fatty acids in colorectal cancer. World J Gastroenterol.

[59] Folch J, Lees M, Sloane Stanley GH (1957). A simple method for the isolation and purification of total lipides from animal tissues. J Biol Chem.

[60] Galanos DS, Kapoulas VM (1965). Preparation and Analysis of Lipid Extracts from Milk and Other Tissues. Biochim Biophys Acta.

[61] Masella R, Vari R, D'Archivio M, Santangelo C, Scazzocchio B, Maggiorella MT, Sernicola L, Titti F, Sanchez M, Di Mario U, Leto G, Giovannini C (2006). Oxidised LDL modulate adipogenesis in 3T3-L1 preadipocytes by affecting the balance between cell proliferation and differentiation. FEBS Lett.

