# Supplementary material for: Visceral fat adipocytes from obese and colorectal cancer subjects exhibit distinct secretory and ω6 polyunsaturated fatty acid profiles and deliver immunosuppressive signals to innate immunity cells
Source: Oncotarget. 2016 Aug 1;7(39):63093–105. doi: 10.18632/oncotarget.10998 (PMC5325349; doi:10.18632/oncotarget.10998)
Supplement: Supplementary file 1 [file oncotarget-07-63093-s001.pdf]

Visceral fat adipocytes from obese and colorectal cancer subjects exhibit distinct secretory and ω6 polyunsaturated fatty acid profiles and deliver immunosuppressive signals to innate immunity cells

Supplementary Materials

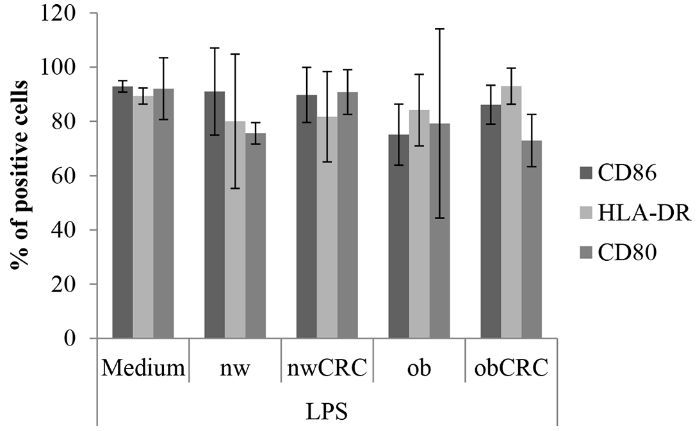

Supplementary Figure S1: Adipocyte microenvironment does not alter DC phenotypic maturation. 5-day-cultured control DC (Medium), nw ($n = 6$), ob ($n = 6$), nwCRC ($n = 4$) and obCRC ($n = 4$) –ACM DC were treated with LPS (10 ng/ml) for 24 hours. Cells were stained with CD80, CD86 and HLA-DR and analyzed by flow cytometry. Data are shown as means of the percentage of positive cells ± SEM.

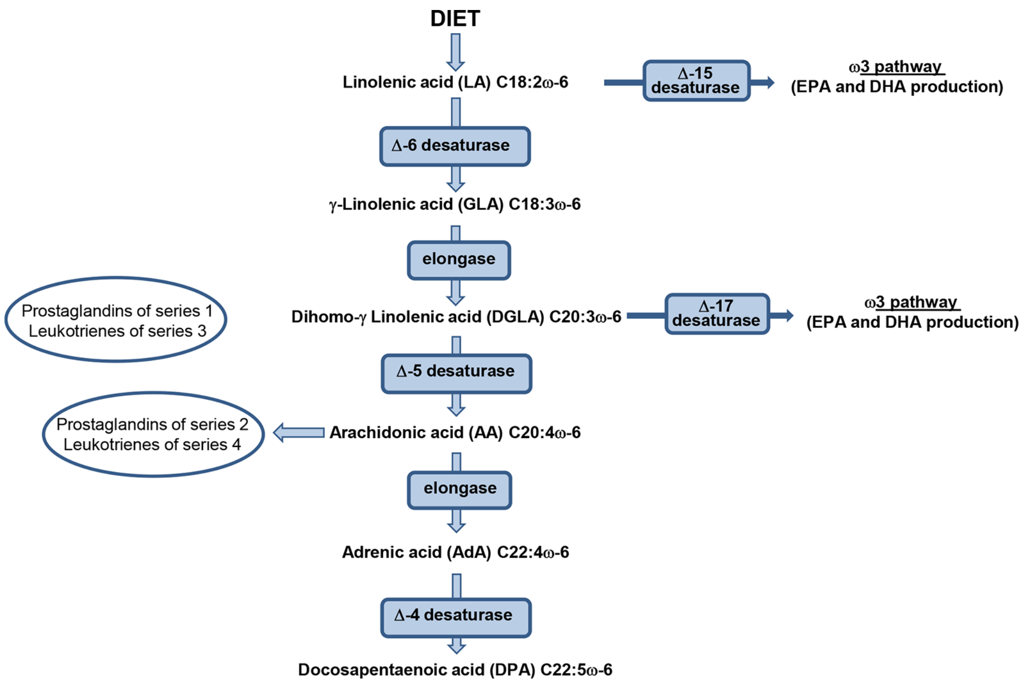

Supplementary Figure S2: Pathway of biosynthesis of ω6 PUFAs. The metabolic pathway of ω6 PUFAs generates different metabolites with pro- or anti-inflammatory activities. The involvement of LA and DGLA in ω3 PUFAs metabolism is indicated.
